# Efficacy and safety of mitoxantrone hydrochloride liposome injection in Chinese patients with advanced breast cancer: a randomized, open-label, active-controlled, single-center, phase II clinical trial

**DOI:** 10.1007/s10637-021-01182-7

**Published:** 2021-10-11

**Authors:** Leiping Wang, Jun Cao, Chunlei Li, Xiaodong Wang, Yannan Zhao, Ting Li, Yiqun Du, Zhonghua Tao, Wenxia Peng, Biyun Wang, Jian Zhang, Sheng Zhang, Zhonghua Wang, Xichun Hu

**Affiliations:** 1 Department of Medical Oncology, Fudan University Shanghai Cancer Center, Shanghai, China; 2 Department of Oncology, Shanghai Medical College, Fudan University, Shanghai, China; 3 Department of Medicine, CSPC Zhongqi Pharmaceutical Technology (Shijiazhuang) Co., Ltd, Shanghai, China

**Keywords:** Breast neoplasms, Mitoxantrone, Liposomes, Efficacy, Safety

## Abstract

*Purpose.* This trial aimed to evaluate the efficacy and safety of mitoxantrone hydrochloride liposome injection (Lipo-MIT) in advanced breast cancer (ABC). *Methods.* In this randomized, open-label, active-controlled, single-center, phase II clinical trial, eligible patients were randomized in a ratio of 1:1 to receive Lipo-MIT or mitoxantrone hydrochloride injection (MIT) intravenously. The primary endpoint was objective response rate (ORR). The secondary endpoints were disease control rate (DCR), progression-free survival (PFS), and safety outcomes. *Results.* Sixty patients were randomized to receive Lipo-MIT or MIT. The ORR was 13.3% (95% confidence interval (CI): 3.8–30.7%) for Lipo-MIT and 6.7% (95% CI: 0.8–22.1%) for MIT. The DCR was 50% (95% CI: 31.3–68.7%) with Lipo-MIT vs. 30% (95% CI: 14.7–49.4%) with MIT. The median PFS was 1.92 months (95% CI: 1.75–3.61) for Lipo-MIT and 1.85 months (95% CI: 1.75–2.02) for MIT. The most common toxicity was myelosuppression. Lipo-MIT resulted in an incidence of 86.7% of leukopenia and 80.0% of neutropenia, which was marginally superior to MIT (96.7% and 96.7%, respectively). Lipo-MIT showed a lower incidence of cardiovascular events (13.3% vs. 20.0%) and increased cardiac troponin T (3.3% vs. 36.7%); but higher incidence of anemia (76.7% vs. 46.7%), skin hyperpigmentation (66.7% vs. 3.3%), and fever (23.3% vs. 10.0%) than MIT. *Conclusions* The clinical benefit parameters of Lipo-MIT and MIT were comparable. Lipo-MIT provided a different toxicity profile, which might be associated with the altered distribution of the drug. Additional study is needed to elucidate the potential benefit of Lipo-MIT in ABC. *Clinical trial registration.* This study is registered with ClinicalTrials.gov (No. NCT02596373) on Nov 4, 2015.

**Supplementary information:**

The online version contains supplementary material available at 10.1007/s10637-021-01182-7.

## Introduction

Breast cancer has become the most frequently diagnosed cancer worldwide according to the latest global cancer data in 2020 [1]. Among females, breast cancer still tops the list of the most common cancer (approximately 2.3 million new cases accounted for 24.5% of all cancer cases among women in 2020) and is still the leading cause of cancer death (about six hundred eighty-four thousand deaths in 2020). Despite advances in early diagnosis, 5–10% of newly diagnosed breast cancer patients were with late-stage presentation and metastasis [2]. In addition, 20–30% of patients with early breast cancer may develop recurrence and metastasis over time [3, 4]. Although metastatic breast cancer remains unlikely to be cured, the meaningful improvement in overall survival was achieved with a median overall survival of three years [5]. Based on molecular profiling, breast cancer could be categorized into three major molecular subtypes: hormone-receptor positive, HER2-positive, and triple-negative breast cancer [6]. In spite of the significant benefit of target therapy and immunotherapy in specific subtypes, chemotherapy is still used as a cornerstone in combination with the other therapies [6, 7]. Chemotherapy is recommended for hormone-insensitive and triple-negative patients with metastatic breast cancer [8]. For HER2-positive patients, the combination of chemotherapy and HER2-targeted agents has shown promising improvement in survival outcomes and was recommended in the ABC 5 guidelines developed by ESO-ESMO [9].

Among many options of chemo-agents, anthracyclines are the most commonly prescribed agents. Mitoxantrone, a synthetic anthracycline anticancer drug, works through inducing DNA lesions, interfering RNA, and inhibiting topoisomerase II to exerting anti-tumor effects [10]. Mitoxantrone-based chemotherapy used to be one of the commonly used chemotherapeutic drugs for breast cancer [11], prostate cancer [12], lymphoma [13], acute leukemia [14], and multiple sclerosis [15] with excellent efficacy. Like many other chemotherapeutic drugs, cardiotoxicity was a major concern for patients treated with mitoxantrone. Several studies have demonstrated the association between mitoxantrone and the increased risk of cardiac dysfunction [16–18]. Due to the black box warnings of cardiotoxicity and a higher risk of developing secondary leukemia, the use of mitoxantrone has been limited, especially in patients who have prior anthracycline therapy [10, 19].

The advantages of liposomal formulation stem from its ability to enhance drug stability, sustain release, and target tumor tissues [10, 20]. Therefore, improved anti-tumor efficacy and reduced toxicity are both expected. The in vivo studies have shown that pegylated liposomal formulation altered the pharmacokinetics and tissue distribution of mitoxantrone, resulting in a more favorable therapeutic response [21, 22]. The previous clinical study demonstrated that much fewer adverse events were observed in the cancer patients in the pegylated mitoxantrone liposome group compared to those using the mitoxantrone at the same dose level, which was a promising improvement in the safety outcomes [23].

The present study was a randomized, open-label, active-controlled, single-center, phase II clinical trial to evaluate the efficacy and safety of mitoxantrone hydrochloride liposome injection (Lipo-MIT) in Chinese patients with ABC who had previously failed to respond to at least two lines of chemotherapies.

## Methods

### Study design

This was a randomized, open-label, active-controlled, single-center, phase II clinical trial conducted in Fudan University Shanghai Cancer Center from Oct 26, 2015, through Apr 6, 2017. The purpose of this study was to assess the efficacy and safety of Lipo-MIT in Chinese patients with ABC. The protocol and the informed consent document were approved by the Ethical Committee of Fudan University Shanghai Cancer Center. The study was conducted in compliance with the principles of Good Clinical Practice and the Declaration of Helsinki. All patients provided the signed informed consent before entering the study. This study was registered in ClinicalTrials.gov: NCT02596373.

The dose selection of Lipo-MIT was based on the results of our phase I study, which demonstrated that Lipo-MIT was safe and effective at 12 to 24 mg/m^2^ [23]. According to the standard treatment protocol of MIT, patients could receive MIT to a maximum cumulative dose of 160 mg/m^2^ to limit the risks of cardiotoxicity, and 120 mg/m^2^ for those who have experienced prior anthracycline therapy [19]. And the conventional treatment consists of 4 to 8 cycles for anthracyclines [24]. To ensure the treatment efficacy, 20 mg/m^2^ was selected as the recommended dose of Lipo-MIT in our study. This dose selection of Lipo-MIT allows patients to take adequate cycles of chemotherapy, and provides operational flexibility for the dose adjustment (to avoid underdosing). Following label instructions, the use of MIT in this study was at 14 mg/m^2^ [25].

After a 28-day screening period (Day -28 to Day -1), eligible patients entered a treatment period (Week 1 to Week 32). They were randomized in a ratio of 1:1 to receive 20 mg/m^2^ of Lipo-MIT (CSPC Zhongqi Pharmaceutical Technology (Shijiazhuang) Co., Ltd.) or 14 mg/m^2^ of mitoxantrone hydrochloride injection (MIT, Sichuan Shenghe Pharmaceutical Co., Ltd.) intravenously, once in a 28-day cycle (up to 8 cycles) until disease progression, intolerable toxicity, or death. According to the safety review, a dose adjustment or treatment postponement was permitted at the discretion of the treating physician. Efficacy and safety were recorded appropriately during the whole study.

### Study population

Female patients who aged 18 to 75 years (inclusive), with histo-pathologically and/or cytologically confirmed ABC and failed for at least two lines of chemotherapy regimens previously, had Eastern Cooperative Oncology Group (ECOG) performance status of 0 to 2, had adequate organ function and bone marrow function, and had at last one measurable lesion with diameter ≥ 10 mm according to Response Evaluation Criteria in Solid Tumors (RECIST, version 1.1) criteria were recruited in this trial. For patients with hormone receptor-positive breast cancer, an endocrine-resistant disease was already developed. Patients with HER2-positive breast cancer were included if they had stopped responding to HER2-targeted therapy or it was not affordable. If patients underwent anthracycline‐containing adjuvant chemotherapy before, a more than 12-month interval was required from the last dose of prior anthracycline chemotherapy to the diagnosis of recurrence.

Main exclusion criteria included: patients with a cumulative dose of doxorubicin (or pirarubicin) > 360 mg/m^2^ or epirubicin > 600 mg/m^2^, previous mitoxantrone treatment, previous anthracycline treatment for recurrence or metastasis, severe uncontrolled diseases, or other second malignancy.

### Efficacy evaluation

The primary endpoint was objective response rate (ORR), which was defined as the proportion of patients who have a complete response (CR) and partial response (PR). Secondary endpoints were disease control rate (DCR), which was defined as the sum of CR, PR, and stable disease (SD) rates; and progression-free survival (PFS) (the time from randomization to disease progression or death). Efficacy evaluation was carried out according to RECIST (version 1.1) criteria.

Efficacy assessment was conducted with appropriate imageological examinations at baseline and every eight weeks during the treatment period. All CRs and PRs required a repeated confirmatory examination four weeks after the initial assessment. After completion of the treatment, PFS follow-up was conducted every three months until documented disease progression or death (for up to 2 years).

### Safety evaluation

Safety evaluation included adverse events (AEs), changes in vital signs, and clinical laboratory tests (such as blood chemistry test, routine blood test, routine urinalysis, electrocardiogram, and echocardiogram). The severity of AEs was assessed according to the National Cancer Institute Common Terminology Criteria for Adverse Events (NCI-CTCAE, version 4.0).

Safety evaluation was conducted from the signing of the informed consent form to 30 days after completion of the treatment. Cardiotoxicity follow-up for patients with dose adjustment due to cardiovascular AEs was performed by echocardiogram once every three months after completion of the treatment (for up to 2 years).

### Statistical analysis

Patients who successfully received at least one dose of study drug with an adequate baseline assessment were included in the full analysis set (FAS); FAS was the primary analysis set for efficacy. Patients who received at least one dose of study drug were included in the safety set (SS); SS was the primary analysis set for safety.

Because of the limited understanding of the new liposomal treatment in Chinese patients, the initial sample size was set at 60 without statistical estimation. The randomization sequences were generated by an independent statistician using SAS software (version 9.4). The sequential numbering method was used for drug allocation.

All statistical analyses were performed using SAS (version 9.4). All statistical tests were 2-tailed at α = 0.05 level for significance. Continuous variables were described by mean, standard deviation, or median (minimum and maximum) values as appropriate, and differences were evaluated by *t*-test or *t’*-test. Categorical or ranked data were described by count and percentage, and differences were evaluated by Chi-square test or Fisher’s exact test. ORR and DCR were summarized for each group along with 95% confidence intervals (CI). Kaplan–Meier analysis was used to estimate the median PFS and the corresponding 95% CI, log-rank test to compare PFS between the two groups, and Cox proportional-hazards model to estimate the hazard ratio and the corresponding 95% CI. Subgroup analysis was conducted according to the molecular subtypes and liver metastases. Adverse events used the Medical Dictionary for Regulatory Activities for coding (MedDRA, version 21.0).

## Results

### Study population and drug administration

Between October 2015 and April 2017, a total of 60 patients were randomized in a ratio of 1:1 to receive 20 mg/m^2^ of Lipo-MIT or 14 mg/m^2^ of MIT intravenously. The CONSORT diagram of the patient’s deposition is shown in Supplementary Fig. 1. The demographics and baseline characteristics of the patients are summarized in Table 1. The important characteristics of patients were well balanced between the two groups. The median age was 56 (range 44–62). The majority of patients (67%) had a hormone-receptor-positive disease, 15% of patients had a triple-negative disease, and 13% had a HER2-positive disease. And all patients had been heavily pretreated; 27 patients in each group had received prior anthracyclines (epirubicin, or doxorubicin) therapy. The baseline average cumulative dose of anthracyclines was 295.8 mg/m^2^ and 288.6 mg/m^2^, without statistical significance between groups (*P* > 0.05) (Table 1).

**Table 1:** Demographics and baseline characteristics

**Characteristic**	**Lip-MIT (N = 30)**	**MIT (N = 30)**	***P*-value**
Sex			
Female, N (%)	30 (100.0)	30 (100.0)	-
Age (years), median (range)	56.0 (27–69)	54.5 (44–62)	0.85
Race or ethnic group,			
Chinese/Han, N (%)	30 (100.0)	30 (100.0)	-
ECOG score, N (%)			1.00
0	0 (0.0)	1 (3.3)	
1	30 (100.0)	29 (96.7)	
Metastatic sites ^a^, n			0.91
Visceral	46	43	
Non-visceral	39	37	
Number of metastatic sites, N (%)			0.27
1	6 (20.0)	6 (20.0)	
2	4 (13.3	9 (30.0)	
3 or more	20 (66.7)	15 (50.0)	
Molecular subtypes, N (%)			0.51
Hormone-receptor positive	17 (56.7)	23 (76.7)	
HER2-positive	5 (16.7)	3 (10.0)	
Triple negative	6 (20.0)	3 (10.0)	
NA	2 (6.7)	1 (3.3)	
Previous oncology therapy, N (%)			
Previous surgery	28 (93.3)	29 (96.7)	0.99
Previous chemotherapy	30 (100.0)	30 (100.0)	
Anthracycline-containing chemotherapy	27 (90.0)	27 (90.0)	
Previous radiation therapy	3 (10.0)	3 (10.0)	
Previous endocrine therapy	23 (76.7)	25 (83.3)	
Baseline cumulative anthracycline dose (mg/m^2^), mean (SD)	295.8 (98.74)	288.6 (115.98)	0.81
History of heart diseases, high blood pressure, diabetes mellitus, or high blood cholesterol, N (%)	5 (16.7)	11 (36.7)	0.26

Data are expressed as counts (percentage) unless otherwise specified

*ECOG* Eastern Cooperative Oncology Group, *Lipo-MIT* mitoxantrone hydrochloride liposome injection, *MIT* mitoxantrone hydrochloride injection, *n* number of sites, *N* number of patients, *SD* standard deviation

^a^A patient may have more than one metastatic site and may have both visceral and non-visceral metastasis

The drug administration information is summarized in Supplementary Table 1. The median number of cycles delivered was two (range 1–6) in the Lipo-MIT group and two (range 1–8) in the MIT group. The percentage of patients who completed four treatment cycles was higher in the Lipo-MIT group (36.7% in the Lipo-MIT group vs. 23.3% in the MIT group).

### Efficacy

**Fig. 1:**
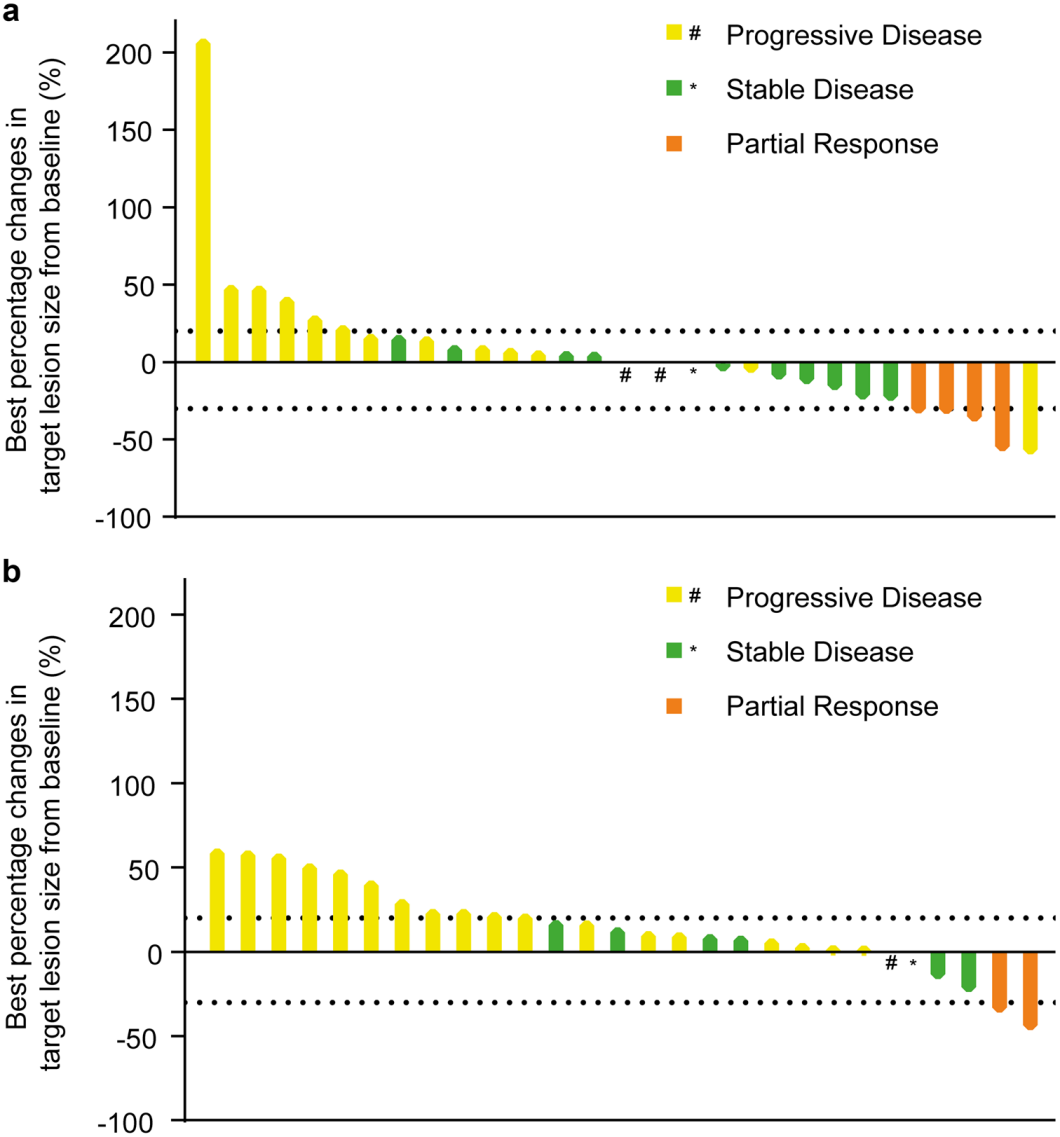
A waterfall plot of the best percent change from baseline in the sum of the diameters of the target lesions. Panel a shows the result of Lipo-MIT group, Panel b shows the result of MIT group

**Fig. 2:**
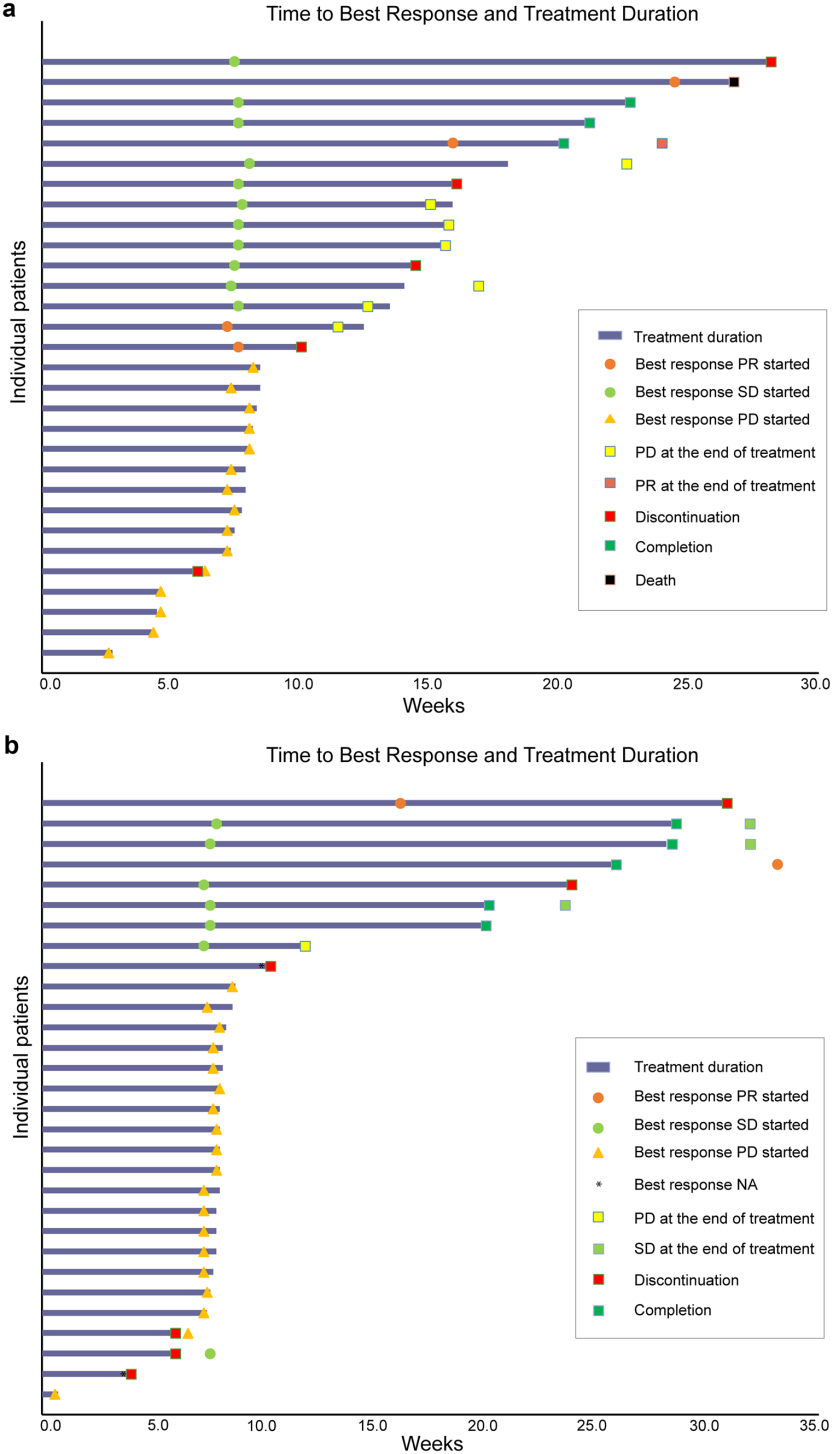
A swimmer plot of the objective responses according to RECIST (version 1.1) from the start of treatment to the end of treatment. Panel a shows the result of Lipo-MIT group, Panel b shows the result of MIT group. Each horizontal bar represents one patient. The treatment duration was defined as the time from the first treatment to the time of documented progression, withdrawal, death, or completion of full course of treatment. PD: progressive disease; PR: partial response; SD: stable disease; NA: not available

All patients in this study were evaluated for response. The best percentage changes from baseline and the best overall response are shown in Fig. 1. Overall, the ORR was 13.3% (95% CI: 3.8–30.7%) and DCR was 50% (95% CI: 31.3–68.7%) in the Lipo-MIT group, with 4 (13.3%) patients achieved PR and 11 (36.7%) SD (Table 2). The ORR in the MIT group was 6.7% (95% CI: 0.8–22.1%) and the DCR was 30% (95% CI: 14.7–49.4%) (Table 2). The ORR favored the Lipo-MIT group, but the difference was not statistically significant (*P* > 0.05). Figure 2 shows a swimmer plot of the time to best responses and the treatment duration. The median PFS was 1.92 (95% CI: 1.75–3.61) months in the Lipo-MIT group versus 1.85 (95% CI: 1.75–2.02) months in the MIT group (Supplementary Fig. 2). The hazard ratio of PFS was 1.13 (95% CI: 0.64–1.99) for the Lipo-MIT group versus the MIT group. No significant difference was found (Log-rank P > 0.05, Supplementary Fig. 2). In subgroup analysis based on the molecular subtypes and liver metastases, we found that the clinical benefit parameters were consistent with the overall result across different subgroups; no statistically significant differences were seen between groups in the response rate and the median PFS (Supplementary Table 2).

**Table 2:** Tumor response

**Efficacy measurement**	**Lipo-MIT**	**MIT**	***P*-value**
Best overall response (%)			0.14
CR	0.0 (0/30)	0.0 (0/30)	
PR	13.3 (4/30)	6.7 (2/30)	
SD	36.7 (11/30)	23.3 (7/30)	
PD	50.0 (15/30)	63.3 (19/30)	
NE	0.0 (0/30)	6.7 (2/30)	
ORR (%)	13.3 (4/30), CI: 3.8–30.7	6.7 (2/30), CI: 0.8–22.1	0.67
DCR (%)	50.0 (15/30), CI: 31.3–68.7	30.0 (9/30), CI: 14.7–49.4	0.11

Data are expressed as percentage (counts) unless otherwise specified. DCR: defined as the percentage of patients with CR or PR or SD. DCR is described by percentage (number of CRs + PRs + SDs / number of patients) and its CI. ORR: defined as the percentage of patients with CR or PR. ORR is described by percentage (number of CRs + PRs / number of patients) and its CI

*CI* 95% confidence interval, *CR* complete response, *DCR* disease control rate, *Lipo-MIT* mitoxantrone hydrochloride liposome injection, *MIT* mitoxantrone hydrochloride injection, *NE* Not evaluable, *ORR* overall response rate, *PD* progress disease, *PR* partial response, *SD* stable disease

### Safety

The summary of AEs is listed in Table 3. Totally, 1751 AEs in 15 system organ classes (SOCs) were reported in 60 enrolled patients, with the most frequent being blood and lymphatic system disorders. All patients in the Lipo-MIT group and the MIT group experienced at least one AE. Grade 3–4 AEs occurred.

**Table 3:** Summary of Adverse Events (AEs)

**AEs **^**a**^	**Lipo-MIT (N = 30)** **N (%)**			**MIT (N = 30)** **N (%)**		
	**All Grades**	**Grade 3**	**Grade 4**	**All Grades**	**Grade 3**	**Grade 4**
Any AEs	30 (100.0)	23 (76.7)	7 (23.3)	30 (100.0)	29 (96.7)	8 (26.7)
Hematological AEs						
Leukopenia	26 (86.7)	15 (50.0)	2 (6.7)	29 (96.7)	16 (53.3)	3 (10.0)
Neutropenia	24 (80.0)	15 (50.0)	3 (10.0)	29 (96.7)	20 (66.7)	8 (26.7)
Anemia	23 (76.7)	6 (20.0)	1 (3.3)	14 (46.7)	2 (6.7)	0 (0.0)
Thrombocytopenia	17 (56.7)	4 (13.3)	5 (16.7)	16 (53.3)	2 (6.7)	0 (0.0)
Non-hematological AEs						
Skin hyperpigmentation	20 (66.7)	0 (0.0)	0 (0.0)	1 (3.3)	0 (0.0)	0 (0.0)
Increased conjugated bilirubin	16 (53.3)	0 (0.0)	2 (6.7)	17 (56.7)	2 (6.7)	0 (0.0)
Increased AST	12 (40.0)	1 (3.3)	0 (0.0)	16 (53.3)	2 (6.7)	0 (0.0)
Increased BNP	10 (33.3)	0 (0.0)	0 (0.0)	10 (33.3)	0 (0.0)	0 (0.0)
Increased total bilirubin	9 (30.0)	1 (3.3)	1 (3.3)	6 (20.0)	0 (0.0)	0 (0.0)
Increased ALT	6 (20.0)	0 (0.0)	0 (0.0)	8 (26.7)	2 (6.7)	0 (0.0)
Increased cTnT	1 (3.3)	0 (0.0)	0 (0.0)	11 (36.7)	0 (0.0)	0 (0.0)
Fever	7 (23.3)	1 (3.3)	0 (0.0)	3 (10.0)	0 (0.0)	0 (0.0)
Fatigue	6 (20.0)	1 (3.3)	0 (0.0)	4 (13.3)	1 (3.3)	0 (0.0)
Hypokalemia	3 (10.0)	1 (3.3)	1 (3.3)	3 (10.0)	0 (0.0)	1 (3.3)
Increased GGT	3 (10.0)	2 (6.7)	0 (0.0)	3 (10.0)	1 (3.3)	0 (0.0)
Hypertension	2 (6.7)	1 (3.3)	0 (0.0)	2 (6.7)	1 (3.3)	0 (0.0)
Pneumonitis	2 (6.7)	1 (3.3)	0 (0.0)	2 (6.7)	1 (3.3)	0 (0.0)
Hypocalcemia	2 (6.7)	1 (3.3)	0 (0.0)	0 (0.0)	0 (0.0)	0 (0.0)
Hyponatremia	2 (6.7)	1 (3.3)	0 (0.0)	0 (0.0)	0 (0.0)	0 (0.0)
Soft tissue infection	2 (6.7)	1 (3.3)	0 (0.0)	0 (0.0)	0 (0.0)	0 (0.0)
Hypophosphatemia	1 (3.3)	0 (0.0)	1 (3.3)	0 (0.0)	0 (0.0)	0 (0.0)
Decreased serum phosphorus	1 (3.3)	1 (3.3)	0 (0.0)	0 (0.0)	0 (0.0)	0 (0.0)
Urethral infection	1 (3.3)	1 (3.3)	0 (0.0)	0 (0.0)	0 (0.0)	0 (0.0)
Chronic bronchitis	0 (0.0)	0 (0.0)	0 (0.0)	1 (3.3)	1 (3.3)	0 (0.0)
Febrile neutropenia	0 (0.0)	0 (0.0)	0 (0.0)	1 (3.3)	1 (3.3)	0 (0.0)

Data are expressed as counts (percentage) unless otherwise specified

*ALT* alanine aminotransferase, *AST* aspartate aminotransferase, *BNP* Brain natriuretic peptide, *cTnT* cardiac troponin T, *GGT* Gamma glutamyl transferase, *Lipo-MIT* mitoxantrone hydrochloride liposome injection, *MIT* mitoxantrone hydrochloride injection, *N* number of patients

^a^Included AEs are adverse events of any grade that occurred in at least 20% of the patients or grade 3–4 adverse events

more frequently in the MIT group compared to the Lipo-MIT group (grade 3–4 AEs reported by 29 patients with MIT, 23 with Lipo-MIT). Eight (26.7%) patients in the Lipo-MIT group discontinued due to an adverse event, while one MIT-treated patient (3.3%) discontinued due to an adverse event. Nine cases of drug-related serious adverse events (SAEs) occurred in six patients in the Lipo-MIT group; four cases of drug-related SAEs occurred in two patients in the MIT group (Supplementary Table 3). One case of death (due to interstitial pneumonia) was reported in the Lipo-MIT group; the causality was assessed as possibly related to the study drug. The details of SAEs are tabulated in Supplementary Table 3.

Overall, myelosuppression was commonly seen in both groups. The lower incidence of leukopenia and neutropenia (all-grade and grade 3–4) was found in the Lipo-MIT group, and the incidence of anemia and thrombocytopenia (all-grade and grade 3–4) was higher in the Lipo-MIT group. Non-hematological AEs were generally mild to moderate compared to hematological AEs. Skin hyperpigmentation was the most common non-hematologic AE in patients treated with Lipo-MIT (grade 1–2: 66.7%) compared to patients with MIT (grade 1–2: 3.3%). Generalized symptoms (fatigue and fever) and the changes of the investigational tests (such as increased conjugated bilirubin, increased aspartate aminotransferase) were reported frequently but generally low-grade in both groups. No increase in the incidence of abnormal liver function was observed in Lipo-MIT group (Table 3).

Cardiovascular AEs were the AEs of special interest in our study. The AEs of cardiac disorders, including ventricular extrasystole, supraventricular extrasystoles, palpitation, sinus tachycardia, and left bundle branch block, were reported by four (13.3%) patients in the Lipo-MIT group, which was lower than that in the MIT group (seven patients, 23.3%). All cardiovascular AEs were grade 1. The elevated cardiac troponin T (cTnT) level, a cardiotoxicity biomarker, was detected in one patient (3.3%) in the Lipo-MIT group, which was much lower than that in the MIT group (11 patients, 36.7%) (Table 3).

## Discussion

Despite the fast advances in treatment options for breast cancer patients, anthracycline-based treatment is still an active and preferable option in clinical practice. The conventional mitoxantrone is effective in the treatment of ABC [26]. In the Chinese Expert Consensus on Anthracyclines Treatment of Breast Cancer [25], the use of anthracyclines alone or in combination is recommended in patients with ABC who are not resistant to anthracycline or have not exceeded the cumulative dose limit. James Neidhart and colleagues reported that the efficacy of mitoxantrone was comparable with doxorubicin in breast cancer patients, with more favorable safety outcomes [27]. However, the usefulness of mitoxantrone was hampered by its cardiotoxicity and myelosuppression.

It is logical to use a liposomal formulation to improve the safety profiles and enhance the anti-tumor effects via permeability and retention effect [28]. A phase II clinical trial of liposomal mitoxantrone was previously conducted in German ABC patients but failed (only 1/17 patients had a PR) [29]. The explanation of this failure from the authors was the drug leaked from the liposomal bilayer and inadequate circulation time [29]. The modifications of the lipid bilayer of the liposome are undoubtedly crucial for the effectiveness of liposomal formulations [30]. Our Lipo-MIT was prepared by the pegylated hydrogenated soy phosphatidylcholine/cholesterol (HSPC/chol) with a good encapsulation efficiency, which was stable over 60 min at 60 °C and over six months at 2–8 °C. The improved circulation time was observed in both the animal model and phase I study [23, 31, 32]. The particle size of Lipo-MIT was down to 60 nm, resulting in a preferential accumulation into tumor tissue in in vivo studies and a fast drug-release rate in in vitro studies [33].

In general, the patients who experienced failure of the first two lines of chemotherapies may have a worse prognosis in the subsequent treatment [34]. In a large retrospective study conducted by Porkka et al., the pooled ORR for the second-line and higher-line treatment was 11% for ABC patients [35]. The ORR (> 2 lines) in our study was 13.3% in the Lipo-MIT group, which was comparable with the previous report. However, we did not observe a significant difference in ORR between the Lipo-MIT and MIT groups (13.3% vs. 6.7%, P > 0.05). Similar results have been seen in other studies on liposomal anthracyclines. A phase III trial showed comparable efficacy of liposomal doxorubicin versus doxorubicin in metastatic breast cancer treatment (ORR were 33% with liposomal doxorubicin and 38% with doxorubicin) [36]. A lower ORR was observed in our study, which was probably due to our inclusion of heavily pretreated patients who failed the prior anthracyclines treatment.

It was worth noticing that the incidence of skin hyperpigmentation was much higher in the Lipo-MIT group than in the MIT group. And the one patient with skin hyperpigmentation in MIT group was assessed as not related to the study drug. In contrast, all the cases of skin hyperpigmentation in the Lipo-MIT group were related to the study drug. However, no patient needed to reduce the dosage, discontinued the medication, or withdrew from the trial due to skin hyperpigmentation. Medical attention and treatments were not needed to resolve this condition. We speculated that the occurrence of skin hyperpigmentation might be related to the characteristics of drug formulation or tissue distribution.

In the previous phase III of liposomal doxorubicin, the risk of cardiotoxicity with liposomal doxorubicin was much lower than that with conventional doxorubicin in patients with metastatic breast cancer [36]. Our safety results were consistent with the previous study by showing a smaller proportion of patients with cardiovascular AEs. And the incidence of increased cTnT was much lower in the Lipo-MIT group than in the MIT group (3.3% vs. 36.7%). The mechanism of reduced cardiotoxicity of liposomal mitoxantrone might be due to the lower peak concentrations [23], larger area under the concentration–time curve [21–23, 33], prolonged half-life [22], and reduced heart tissue distribution [22]. Myelosuppression was frequently seen in our study. Overall, the incidence of hematological AEs was similar between groups. But more hematological SAEs were reported in the Lipo-MIT group (seven cases with Lipo-MIT, one case with MIT). The comparative safety of Lipo-MIT and MIT was still inconclusive.

One major limitation of our study was the small sample size. Because this was the first study to evaluate the efficacy and safety of Lipo-MIT in Chinese patients with ABC, the sample size was determined without any statistical estimation. Several improvements with Lipo-MIT in efficacy endpoints and safety outcomes were not statistically significant due to the effects of small sample size.

In conclusion, this study provided additional information about the Lipo-MIT in the ABC patients, which indicated a potential advantage in efficacy and cardiovascular safety. But a statistically significant difference of Lipo-MIT over MIT in ABC was not established in this study. It is worthwhile to further elucidate the risk and benefit of Lipo-MIT as an alternative to MIT in ABC setting.

## Supplementary Information

Below is the link to the electronic supplementary material.

Supplementary file1 (DOCX 24 KB)

Supplementary file2 (DOC 219 KB)

## Data Availability

The datasets generated during and/or analyzed during the current study are available from the corresponding author on reasonable request.

## Abbreviations

ABC: Advanced breast cancer
AEs: Adverse events
CR: Complete response
cTnT: Cardiac troponin T
DCR: Disease control rate
ECOG: Eastern Cooperative Oncology Group
FAS: Full analysis set
Lipo-MIT: Mitoxantrone hydrochloride liposome injection
MIT: Mitoxantrone hydrochloride injection
ORR: Objective response rate
PD: Progressive disease
PFS: Progression-free survival
PR: Partial response
SD: Stable disease
SS: Safety set

## Code availability

All statistical analyses were performed using SAS software (version 9.4).
