# Supplementary material for: Efficacy and safety of mitoxantrone hydrochloride liposome injection in Chinese patients with advanced breast cancer: a randomized, open-label, active-controlled, single-center, phase II clinical trial
Source: Invest New Drugs. 2021 Oct 11;40(2):330–9. doi: 10.1007/s10637-021-01182-7 (PMC8993786; doi:10.1007/s10637-021-01182-7)
Supplement: Supplementary file 1 — Supplementary file1 (DOCX 24 KB) [file 10637_2021_1182_MOESM1_ESM.docx]

**Supplementary Table 1** Drug Administration

|  | **Lip-MIT (N = 30)** | **MIT (N = 30)** |
| --- | --- | --- |
| Median number of cycles (range) | 2 (1-6) | 2 (1-8) |
| Median treatment duration in weeks (range) | 9.21 (2.71-28.14) | 8.00 (0.71-30.86) |
| Median administered dose per cycle in mg (range) | 31.6 (24.9-36.6) | 22.5 (14.8-28.4) |
| Median Cumulative dose administered per person in mg (range) | 69.0 (29.4-217.2) | 46.4 (16.9-188.0) |
| Percentage of patients receiving 4 cycles of treatment (count) | 36.7% (11) | 23.3% (7) |

Lipo-MIT: mitoxantrone hydrochloride liposome injection; MIT: mitoxantrone hydrochloride injection; N: number of patients.

**Supplementary Table 2** Efficacy Outcomes Stratified by Molecular Subtypes and Liver Metastases

| **Subtypes** | **ORR (%)** | | **DCR (%)** | | **Median PFS (months)** | |
| --- | --- | --- | --- | --- | --- | --- |
|  | **Lipo-MIT** | **MIT** | **Lipo-MIT** | **MIT** | **Lipo-MIT** | **MIT** |
| Molecular subtypes | | | | | | |
| Hormone-receptor positive | 11.8 (2/17),  CI:1.5-36.4 | 4.3 (1/23),  CI:0.1-21.9 | 41.2 (7/17), CI:18.4-67.1 | 30.4 (7/23), CI:13.2-52.9 | 1.88,  CI: 1.49-6.31 | 1.85,  CI: 1.75-2.41 |
| HER2-positive | 20.0 (1/5),  CI:0.5-71.6 | 0.0 (0/3),  CI:29.2-100.0 | 80.0 (4/5),  CI:28.4-99.5 | 33.3 (1/3),  CI:0.8-90.6 | 3.50,  CI: 1.88-NA | 1.92,  CI: 1.09-NA |
| Triple negative | 0.0 (0/6),  CI:54.1-100.0 | 33.3 (1/3),  CI:0.8, 90.6 | 50.0 (3/6),  CI:11.8, 88.2 | 33.3 (1/3),  CI:0.8, 90.6 | 2.33,  CI: 1.06-NA | 2.02,  CI: 1.82-NA |
| Liver metastases | | | | | | |
| Without LM | 6.7 (1/15),  CI:0.2-31.9 | 6.7 (1/15),  CI:0.2-31.9 | 66.7 (10/15),  CI:38.4-88.2 | 40.0 (6/15),  CI:16.3-67.7 | 3.67,  CI:1.72-8.40 | 2.02,  CI:1.75-7.83 |
| With LM | 20.0 (3/15),  CI:4.3-48.1 | 6.7 (1/15),  CI:0.2-31.9 | 33.3 (5/15),  CI:11.8-61.6 | 20.0 (3/15),  CI:4.3-48.1 | 1.88,  CI:1.19-2.68 | 1.82,  CI:1.72-1.88 |

ORR and DCR are expressed as percentage (counts) with 95% confidence interval. PFS is expressed in months with 95% confidence interval. DCR: defined as the percentage of patients with CR or PR or SD. DCR is described by percentage (number of CRs+PRs+SDs / number of patients) and its CI. ORR: defined as the percentage of patients with CR or PR. ORR is described by percentage (number of CRs+PRs / number of patients) and its CI.

CI: 95% confidence interval; CR: complete response; DCR: disease control rate; NA: not available; ORR: overall response rate; PFS: progression-free survival; PR: partial response; SD: stable disease. LM: Liver metastases; Lipo-MIT: mitoxantrone hydrochloride liposome injection; MIT: mitoxantrone hydrochloride injection.

**Supplementary Table 3** Summary of Serious Adverse Events (SAEs)

| **SAEs** | **Lipo-MIT**  **(N = 30) N (%)** | **MIT**  **(N = 30) N (%)** |
| --- | --- | --- |
| Hematological SAEs |  |  |
| Thrombocytopenia | 4 (13.3) | 1 (3.3) |
| Leukopenia | 2 (6.7) | 0 (0.0) |
| Neutropenia | 1 (3.3) | 0 (0.0) |
| Non-hematological SAEs |  |  |
| Soft tissue infection | 1 (3.3) | 0 (0.0) |
| Interstitial pneumonia | 1 (3.3) | 0 (0.0) |
| Pneumonitis ^a^ | 0 (0.0) | 2 (6.7) |
| Chronic bronchitis | 0 (0.0) | 1 (3.3) |

Data are expressed as counts (percentage) unless otherwise specified.

^a^ Pneumonitis included one case of noninfectious pulmonary inflammation and one case of pneumonia.

Lipo-MIT: mitoxantrone hydrochloride liposome injection; MIT: mitoxantrone hydrochloride injection; N: number of patients.
